# A Framework Based on Reference Data with Superordinate Accuracy for the Quality Analysis of Terrestrial Laser Scanning-Based Multi-Sensor-Systems

**DOI:** 10.3390/s17081886

**Published:** 2017-08-16

**Authors:** Ulrich Stenz, Jens Hartmann, Jens-André Paffenholz, Ingo Neumann

**Affiliations:** Geodetic Institute, Leibniz Universität Hannover, Nienburger Str. 1, 30167 Hannover, Germany; hartmann@gih.uni-hannover.de (J.H.); paffenholz@gih.uni-hannover.de (J.-A.P.); neumann@gih.uni-hannover.de (I.N.)

**Keywords:** TLS, quality analysis, multi-sensor-systems, accuracy, calibration, laser tracker, backward modelling

## Abstract

Terrestrial laser scanning (TLS) is an efficient solution to collect large-scale data. The efficiency can be increased by combining TLS with additional sensors in a TLS-based multi-sensor-system (MSS). The uncertainty of scanned points is not homogenous and depends on many different influencing factors. These include the sensor properties, referencing, scan geometry (e.g., distance and angle of incidence), environmental conditions (e.g., atmospheric conditions) and the scanned object (e.g., material, color and reflectance, etc.). The paper presents methods, infrastructure and results for the validation of the suitability of TLS and TLS-based MSS. Main aspects are the backward modelling of the uncertainty on the basis of reference data (e.g., point clouds) with superordinate accuracy and the appropriation of a suitable environment/infrastructure (e.g., the calibration process of the targets for the registration of laser scanner and laser tracker data in a common coordinate system with high accuracy) In this context superordinate accuracy means that the accuracy of the acquired reference data is better by a factor of 10 than the data of the validated TLS and TLS-based MSS. These aspects play an important role in engineering geodesy, where the aimed accuracy lies in a range of a few mm or less.

## 1. Introduction

Thanks to its high data acquisition rate, Terrestrial laser scanning (TLS) is an appropriate measurement method for many tasks in engineering geodesy. In addition to standard application fields like documentation or 3D- modelling of objects, TLS has been developed in areas like monitoring or industrial surveying. The wider range of applications is strongly connected with increasing requirements in terms of accuracy and efficiency. These requirements have to undergo a process of quality analysis to verify common quality measures (precision, accuracy, resolution, sensitivity, etc.). Regarding accuracy the quality of measurements can be described quantitatively by the measurement uncertainty. Therefore quality criteria have to be established and evaluated by implementation of suitable quality parameters [1]. For the estimation of measurements uncertainty the international Guide to the Expression of Uncertainty of Measurements (GUM) standard has been defined [2]. Applications of GUM in the field of TLS are described e.g., in [3,4]. The goal of this paper was the definition of a framework and process to optimally define a quality assurance and quality analysis based on reference data.

**Figure 1:**
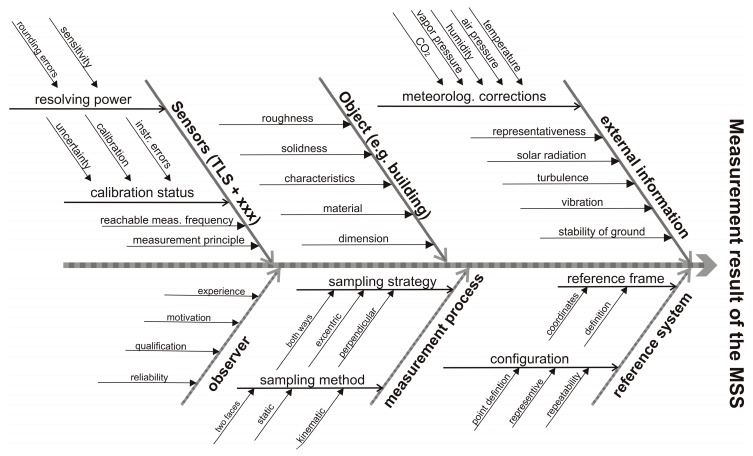
Influences on the measurements with Terrestrial laser scanning (TLS) and TLS-based multi-sensor-system (MSS). Modified from [8].

In order to increase accuracy and efficiency different approaches are made and documented in literature. Multi-sensor-system (MSS) are designed to meet this requirements. They profit from the benefits of complementary sensors and usually kinematic MSS are characterized by a higher data acquisition rate and a better adaption relating to the scan geometry. MSS can be defined as a combination of complementary sensors integrated on a common sensor platform, or a combination of sensors linked by their measurements [5]. Due to the participation of different sensors the quality assurance in the area of MSS is a multi-level process. An introduction and motivation regarding the necessity of quality assurance for MSS is given in [5]. Quality analysis as well as the validation of quality parameters, e.g., correctness, precision, accuracy and integrity are fundamental aspects of quality assurance. Examination of the MSS literature shows different methods of analyzing TLS and TLS- based MSS. An intensity-based approach to determine the precision of point clouds without separating different influence factors for TLS was described in [6,7]. An overview of the occurring influence factors is shown in the following Ishikawa diagram (Figure 1). The diagram is a sufficient way to visualize and explore the influences and their impact on TLS-based measurements. Furthermore, causes that have adverse effects on the measurements can be identified. These effects are systematically assigned and one can focus on the main influencing factors.

These influencing factors can be divided into four main groups:

- Sensor-specific influences
- Influences dependent on the captured object
- Influences caused by the configuration of the measurement or the measurement process
- External influences like the atmospheric conditions

Sensor-specific influences like axis deviations [9,10,11,12,13], object-specific influences like material, colour and reflectance [14] and distance and incidence angle-caused influences are investigated separately and documented in the literature [15,16,17]. Approaches to combine all influences in one model are made in the context of forward modelling based on Monte Carlo Simulations in [18]. In the following an approach based on the comparison of points or point clouds and points or point clouds with superordinate accuracy (reference points/clouds) for the validation of quality parameters is described (backward modelling) [19]. In this context superordinate accuracy means that the accuracy of the acquired reference data is better by a factor of 10 than the data of the validated TLS and TLS-based MSS.

The derivation of quality parameters results from the differences between the captured data and the reference data. This approach should be generally valid for most kinds of sensors (pointwise- and area-based sensors) as well as MSS (kinematic and static). For a full quality assurance the combination of forward and backward modelling should be a promising strategy. To guarantee the accuracy of MSS several validation steps are necessary. A general overview of approaches referring to cloud to cloud analysis based on real measurement data is shown in [20]. Here a basic distinction can be established between geometry-based and point cloud-based modelling procedures for deformation analysis tasks [21].

In a first step the relevant influence factors for TLS and TLS-based MSS have to be identified. In a mostly automated process like TLS the observer has a minor impact. Under stable laboratory conditions external parameters like the atmospheric conditions are usually negligible. Relevant influences are the characteristics of the object (e.g., material and colour), the measurement configuration (e.g., angle of incidence and distance) and the sensors used. As shown in Figure 1 the calibration and the calibration status of the sensors and especially the MSS sensor integration and synchronization, have a significant effect on the measurement results. For this reason it is a very complex process.

For the abovementioned quality assurance in the backward modelling approach, a suitable environment is necessary. This environment must meet different requirements which are listed and described in the next section.

## 2. Environment/Infrastructure

As mentioned before, a suitable environment has to be provided (Table 1). These environments must meet requirements like stable atmospheric (laboratory) conditions, a high accurate reference frame for the referencing of all participating sensors, targets for each kind of sensor to provide the reference points and reference geometries with different colors and materials.

One should try to find a relevant test scenario for all significant influence factors from Figure 1. Sensors with superordinate accuracy must be available to capture the reference data.

The various components and their practical implementation at the Geodetic Institute Hanover (GIH) are described in the following sections.

### 2.1. 3D-Laboratory and 3D-Reference Frame

To perform a sensor calibration or backward modelling laboratory conditions are recommended in order to minimize external effects. To guarantee a common reference frame for area-based and point-based measurements, targets for both sensor types must be available. All target types have to fit in a single type of target mounts, which represent the reference points. The reference geometries and all participating sensors must be referenced within this frame, using the reference points, to ensure the comparability of all captured data. The accuracy of the reference frame must be at least by a factor of three better than the accuracy of the investigated sensors or MSS. Therefore the reference frame has to be determined by a sensor with superordinate accuracy from different standpoints. Afterwards a bundle block adjustment has to be performed. Furthermore, all participating sensors and targets have to be checked and calibrated by the operator or the manufacturer.

**Figure 2:**
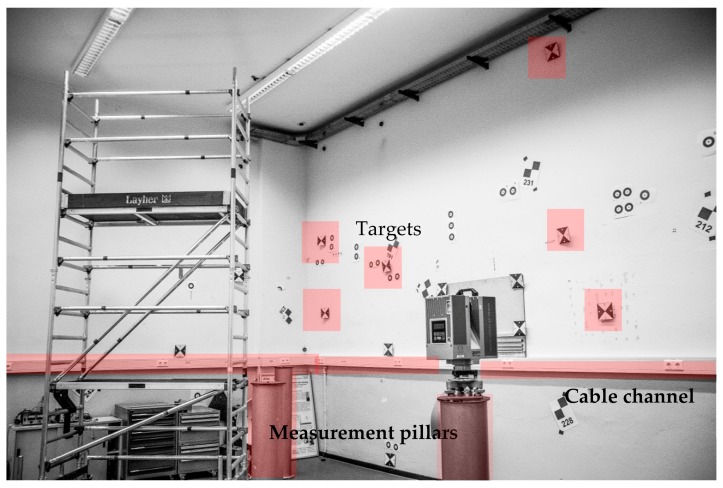
3D-laboratory of the Geodetic Institute Hanover (GIH) with measurement pillars and cable channel.

**Figure 3:**
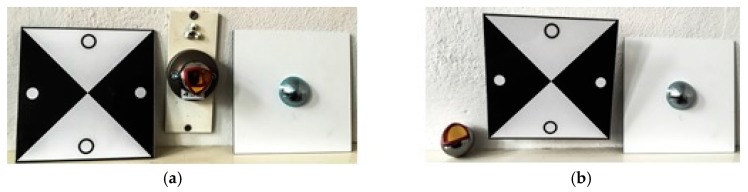
Target mount for the combined adaption of the targets and corner cube reflectors (CCRs). (**a**) target front, target mount with CCR, target back; (**b**) CCR, target mount with target, target back.

A practical implementation that meets these requirements is shown in Figure 2 and Figure 3. Laboratory conditions, especially stable atmospheric conditions, are realized in the 3D-laboratory of the GIH (Figure 2). The dimensions of the room (length × width × height) are 8.8 m × 6.4 m × 4.8 m. For some sensors/MSS the size of the laboratory is maybe too small, but this paper focuses on the general strategy and process which is transferable to larger laboratories. The reference system is represented by 56 installed magnetic target mounts and six measurement pillars.

The target mounts could be equipped with corner cube reflectors (CCRs) for laser tracker measurements or targets for surface based sensors like TLS or photogrammetric camera systems (Figure 3). The targets for the area based sensors are manufactured in-house, so that there are no accuracy specifications available. To receive this information the targets have to undergo a calibration process which is described in Section 3.

**Figure 4:**
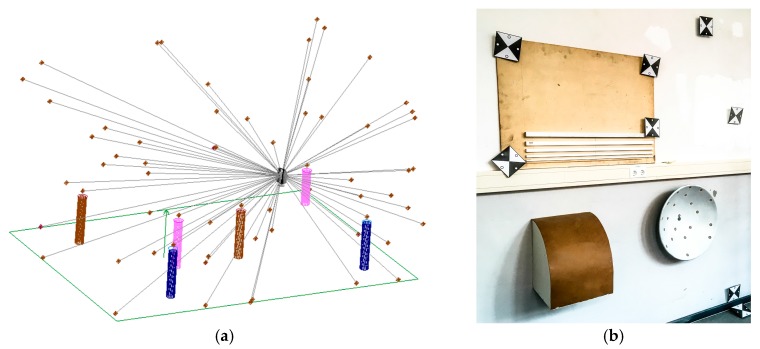
(**a**) Determination of the reference points coordinates with Spatial Analyzer for the visibility analysis; (**b**) reference geometries (cuboids, curved surface and paraboloid).

From three different stand points the x-, y- and z-coordinates of the reference points are determined by laser tracker measurements. In preparation of the measurements a visibility analysis for each stand point was processed with Spatial Analyzer (New River Kinematics, Williamsburg, VA, USA) to ensure that each reference point is measurable (Figure 4a). Afterwards the bundle block adjustment was processed. The standard deviation as parameter for the accuracy of the reference points after adjustment amounts to 0.05 mm.

### 2.2. Reference Geometries

Additionally to the reference points, different reference geometries have to be installed (Figure 4b). These geometries consist of different materials (wood, metal and plastic), colours and shapes (cuboid, paraboloid and curved surface). The variations among the reference geometries have to cover all influences factors described in Section 1. Table 2 provides an overview of all reference geometries regarding to their sensitive influencing factors.

As point-based references the targets shown in Figure 3 were used. For sensors like TLS where no discrete point measurement is possible, the estimation of the x-, y- and z-coordinates of the target centre, as a single point, is shown in Section 3.1.

### 2.3. Sensors with Superordinate Accuracy/Investigated Sensors and MSS

**Figure 5:**
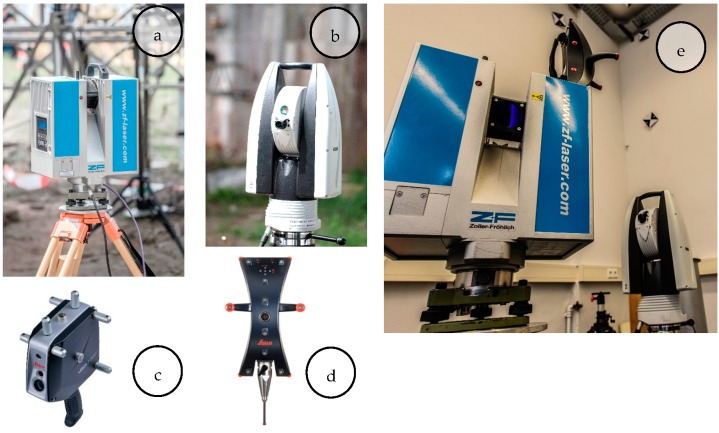
Participating sensors: (**a**) Z+F Imager 5006; (**b**) Leica AT960 LR; (**c**) Leica T-Scan 5 (metrology.leica-geosystems.com); (**d**) Leica T-Probe (www.hexagonmi.com); (**e**) MSS (kinematic-TLS (k-TLS)) consisting of laser scanner Z+F Imager 5006, Leica T-Probe, Leica Absolute Tracker 960 Long Range (AT960 LR).

The participating sensors (Figure 5) can be divided into two classes. On the one hand there are sensors with superordinate accuracy and high resolution to establish the reference frame, capture the reference data and reference the validated sensors. On the other hand there are the investigated sensors which capture the reference geometries with less accuracy (Table 3).

In a practical realization, the reference data was captured with the Leica T-Scan 5 (Leica Geosystems AG, Heerbrugg, St. Gallen, Switzerland, Figure 5c, [22] referenced by the Leica AT 960 LR (Figure 5b, [23]). The investigated sensors are a TLS (Zoller+Fröhlich (Z+F) Imager 5006, (Zoller & Fröhlich GmbH, Wangen, Baden-Wurttemberg, Germany, Figure 5a, [24]) in static mode (s-TLS) and in combination with the Leica T-Probe (Figure 5d) also referenced by Leica AT960 LR as MSS (Figure 5e) in kinematic mode (k-TLS) [25].

**Figure 6:**
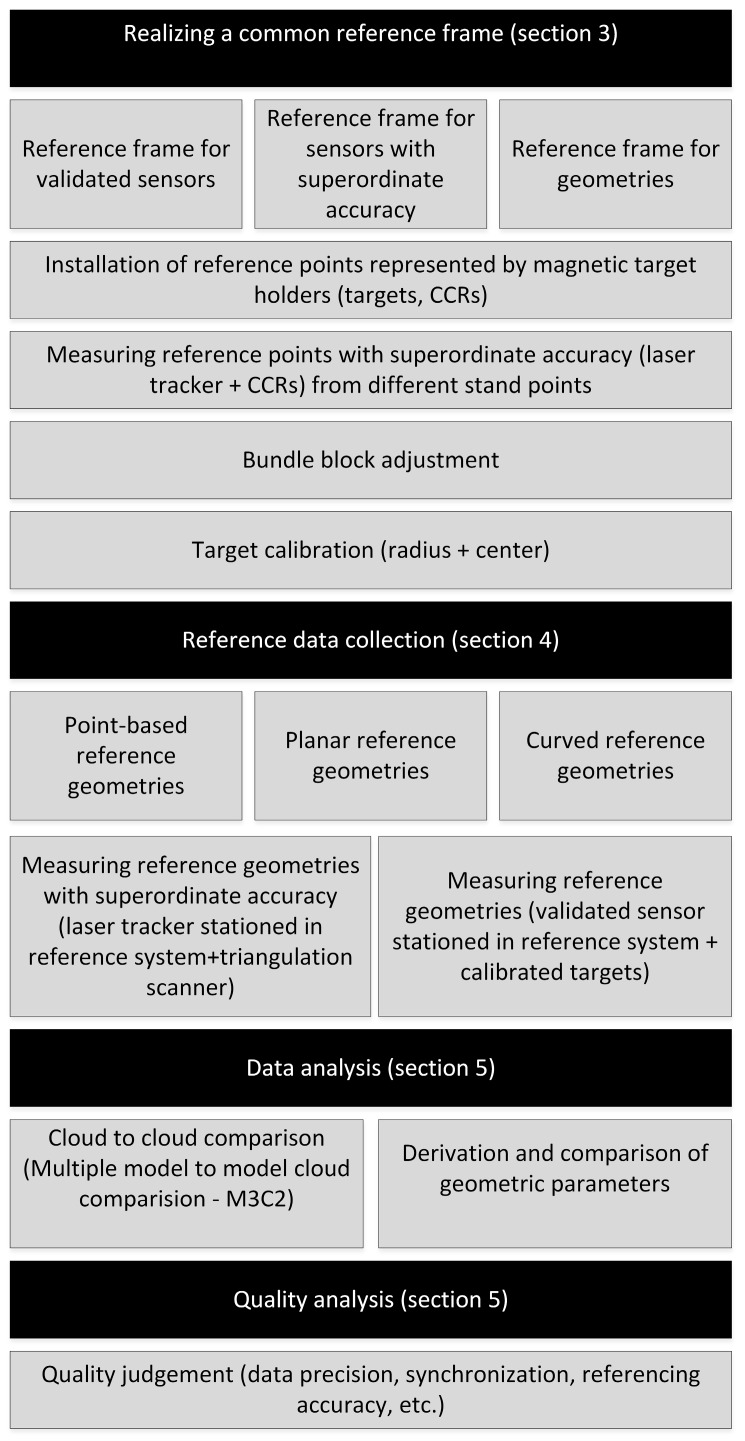
Validation process (workflow).

Under the discribed envirometal conditions, the process of sensor and MSS validation is performed. The single steps of the whole workflow are shown in Figure 6 and will be explained in the following sections.

## 3. Realization of a Reference Frame for Sensors and Geometries

As described in Section 2.1, the targets representing the reference points require accuracy information which is estimated through the following calibration process. Two parameter have to be determined: the targets’ centre (Section 3.1) and the targets’ radius (Section 3.2). The target radius has to match half the diameter of a 1.5” CCR.

Additionally, the target centre should be identical to the CCR centre. To meet these requirements the calibration process is carried out in two steps. These two steps have to be performed for each of the 39 targets used here. In addition, point-like references are generated by this process.

### 3.1. Calibration of the Target Centre

The calibration of the targets is realized in two different measuring setups. In the first set-up, a target is placed in the target holder and scanned in eight different positions with the Leica T-Scan 5. The first position of the target can be set arbitrarily.

**Figure 7:**
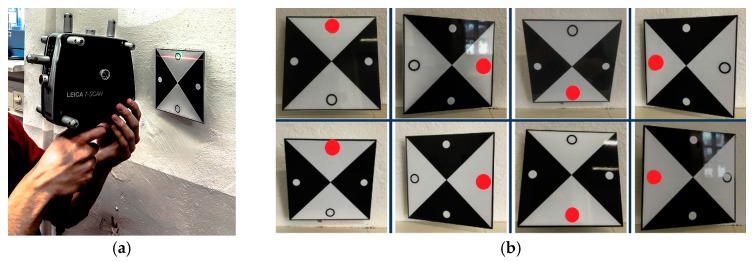
(**a**) Scan of each target with Leica T-Scan 5 for the determination of the target centre and (**b**) the eight scan positions.

To calibrate the targets, all targets should be mounted in the same target holder (Figure 7a). In this case a target holder in height of the tilt axis of the laser tracker is used. The accuracy of the captured coordinates depends on the orientation of the T-Scan, the object surface and the reflector orientation to the laser beam. Therefore, the laser tracker is positioned perpendicular to the target surface. The distance between target and laser tracker was approximately two meters, so the tracker accuracy can be estimated with 27 μm (15 μm + 6 μm/m MPE) [22].

**Figure 8:**
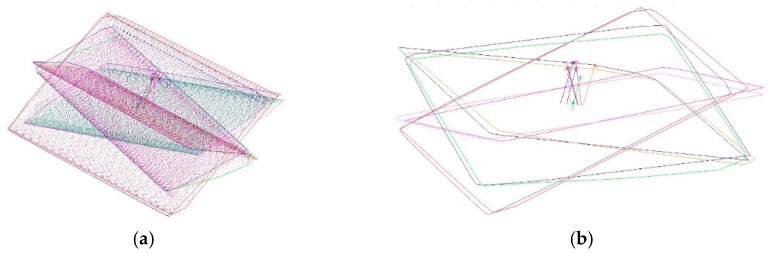
(**a**) Scanned; (**b**) estimated planes and intersection points.

Then, the target panel is rotated by 90° clockwise. According to the first four positions, the target is tilted from the initial into the exactly opposite position and turned clockwise in 90° steps again (Figure 7b). In result eight point clouds for each target were detected with an uncertainty, according to the manufacturer, of ±80 μm + 3 μm/m (2σ for flat surfaces) [22]. Through each point cloud a plane (p1–p8) is estimated (Figure 8) with a fit tolerance of 50 μm using Spatial Analyzer. Before this, all points that lie on the wall plane must be deleted in order not to distort the estimation results.

**Figure 9:**
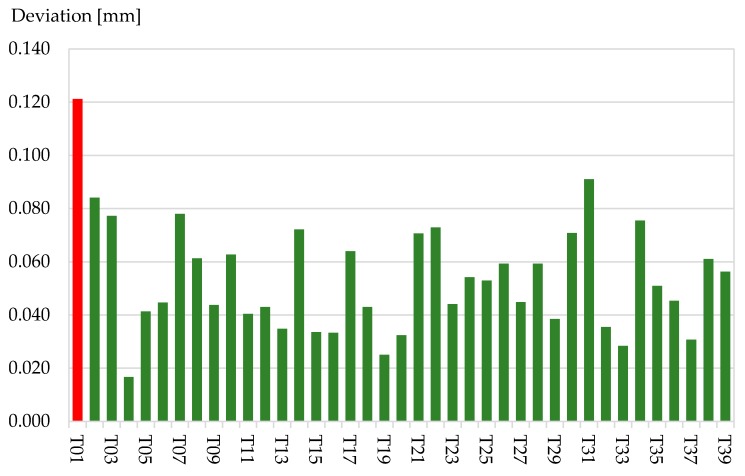
Deviation of target centre to reference point measured with laser tracker on CCR for the targets T01–T39 in [mm].

For a selected number of combinations of three planes (p1p2p3, p1p2p4, p1p3p4, p2p3p4, p5p6p7, p5p6p8, p5p7p8 and p6p7p8) the intersection point was calculated. Ultimately the Euclidean distance between the centre of gravity for the eight intersection points and the reference point was calculated. For all targets except target T01 the Euclidean distance is minor than 100 μm (Figure 9). The standard deviation for the estimation of the targets centre is 0.021 mm. The mean value of the distance between the calculated target centre and the centre of the reference point measured with CCR is 0.054 mm.

### 3.2. Calibration of the Target Radius

**Figure 10:**
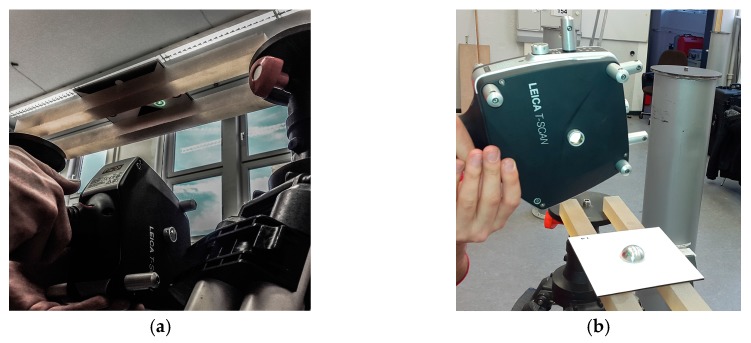
Scan of each targets (**a**) front side and (**b**) backside with Leica T-Scan 5 for the determination of the target radius.

In the second step, the targets are placed on two Zerodur bars (horizontally aligned on two industrial tripods) in a position that allows scanning the back and the front side of the target (Figure 10).

**Figure 11:**
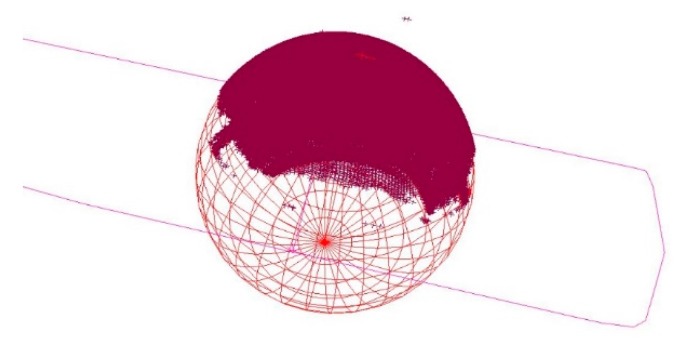
Fitted plane through targets front side and sphere to targets backside.

The laser tracker was again stationed at a distance of two meters. In this setup both the front and the backside was scanned with the Leica T-Scan 5 delivering two high accurate and high resolution point clouds for each of the 39 targets. Points not belonging to the reduced half sphere on the backside and the plane on the front side were deleted. Afterwards a sphere is fitted to the point cloud of the targets backside and a plane to the front side using Spatial Analyzer (Figure 11). The fit tolerance is again set to 50 μm.

**Figure 12:**
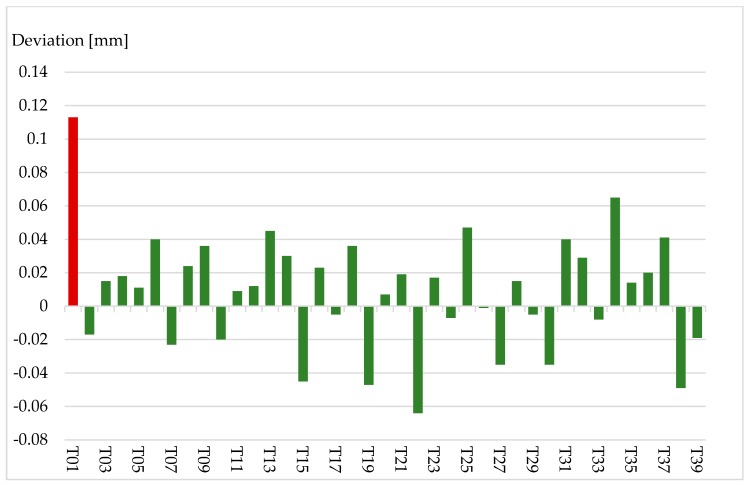
Deviation of target radius to CCR-radius for the targets T1–T39 in [mm].

According to the manufacturer datasheet the measurement uncertainty for spherical radii is specified to ±50 μm (MPE) at less than 8.5 m [22]. The centre of the fitted sphere should theoretically lie in the fitted plane of the front side and should have the same radius like a 1.5” CCR (0.75”/19.05 mm). Figure 12 shows the differences between CCR radius and estimated radius of the targets related to the centre on the targets front side. For 38 of the 39 targets the difference is less than 100 μm. The standard deviation for the estimation of the targets radius is 0.034 mm. The mean value for the difference of targets radius to CCR- radius is 0.009 mm. Again, only target T01 shows less accuracy and will not be used for further measurements.

## 4. Data Sampling for Areal Reference Geometries

In preparation of the measurements the participating sensors have to undergo a calibration process. Especially the kinematic MSS have to be calibrated carefully to guarantee a correct temporal and spatial integration of the individual sensors. The literature shows different approaches for calibration procedures for TLS [26] and the synchronization [27] and calibration of TLS based MSS. An overview of different calibration procedures can be found in [21].

In the reported work, a plane-based calibration process according to [28] was performed. The estimation of the six degrees of freedom (DOF) for the calibration of this specific MSS is described in details in [25,29]. The T-Scan 5 undergoes the manufacturer calibration process using the supplied calibration sphere.

**Figure 13:**
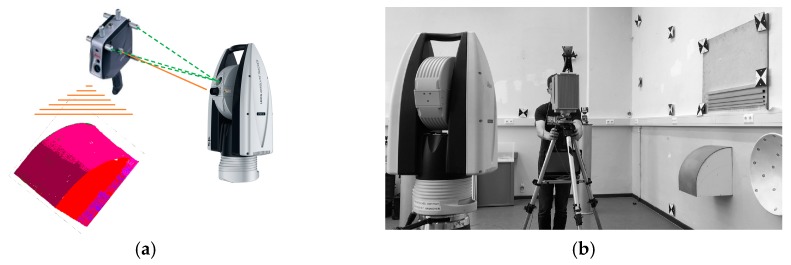
Scan of reference geometries using (**a**) Leica T-Scan 5 and AT960 LR and (**b**) MSS.

**Figure 14:**
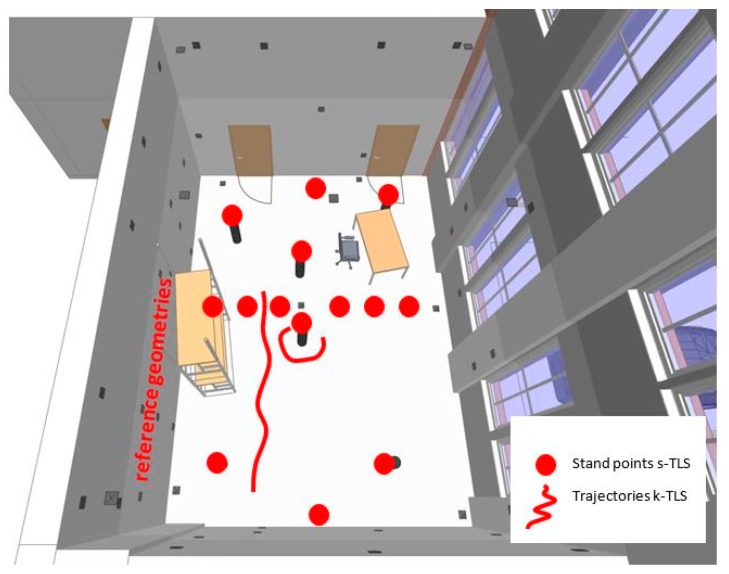
Trajectories of k-TLS and stand points for s-TLS.

After calibration the data of the reference geometries (Section 2.2) was captured in three steps. First step is generating the reference data with the T-Scan 5 referenced by the AT960 LR (Figure 13a). The second step is the measurement of the reference geometries with s-TLS (Z+F Imager5006) and in a third step with k-TLS (MSS, Section 2.3, Figure 13b). The measurements took place under following conditions: two persons, 50 h per person, 35 static scans, seven kinematic scans as well as scans of all reference geometries, pillars, pipes and cable channels with the T-Scan 5. With the k-TLS-based MSS (Figure 5e) the reference geometries were captured with a rotational speed of the vertical mirror of the laser scanner of 50 Hz from a distance of approx. 2 m with a speed of 0.07 m/s. The trajectories for the k-TLS and the stand points for the s-TLS are shown in Figure 14.

As result of the measurements reference point clouds of the geometries are captured with very high resolution (the total number of points for each geometry is shown in Table 4) and accuracy with T-Scan 5. Point clouds with lower resolution and lower precision were captured with the investigated sensors and MSS.

As mentioned in Section 1, different approaches of cloud comparison are shown in [20,30]. In general, a subdivision in direct cloud to cloud comparison, cloud to mesh comparison, mesh to mesh comparison and comparison of geometric parameters derived from the point clouds can be made.

In the following section two concepts are presented:

- The direct point cloud to point cloud comparison using the M3C2-algorithm (multiple scale model to model cloud comparison, [31].
- The comparison of geometrical parameters derived from the point clouds, like the cylinders diameter or the focal length of the paraboloid.

## 5. Data Analysis

To validate the quality of the investigated sensors point clouds, geometrical parameters of the reference geometries were derived from the point clouds with superordinate accuracy and compared to the point clouds of s-TLS and k-TLS. Section 5.1 presents two examples with the comparison of the focal length of a paraboloid and the diameter of the a cylinder. In Section 5.2 the M3C2-distance for different point clouds were calculated exemplarily. Further results and comparisons and a more refined analysis process are part of an additional paper.

### 5.1. Comparison of Geometrical Parameters

**Figure 15:**
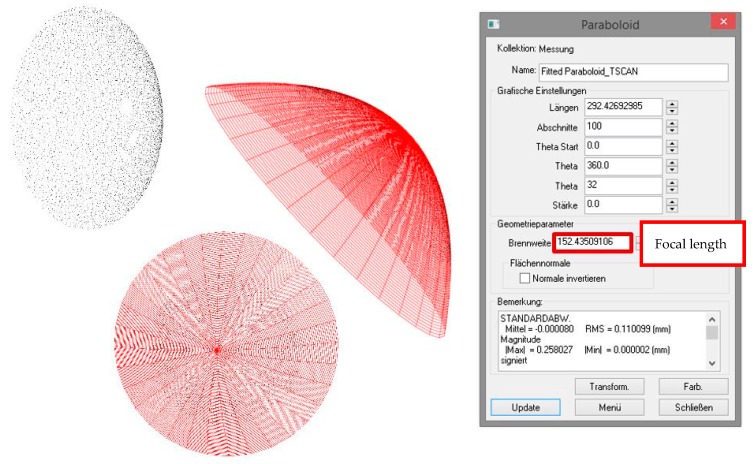
Focal length of paraboloid in mm derived from T-Scan 5 measurements and calculated with Spatial Analyzer.

To estimate geometric parameters in a first step, the geometry must be fitted to the point cloud. In case of the paraboloid Spatial Analyzer was used (Figure 15) to fit the paraboloid and estimate its focal length for the point cloud with superordinate accuracy and the point clouds of both investigated sensors.

**Figure 16:**
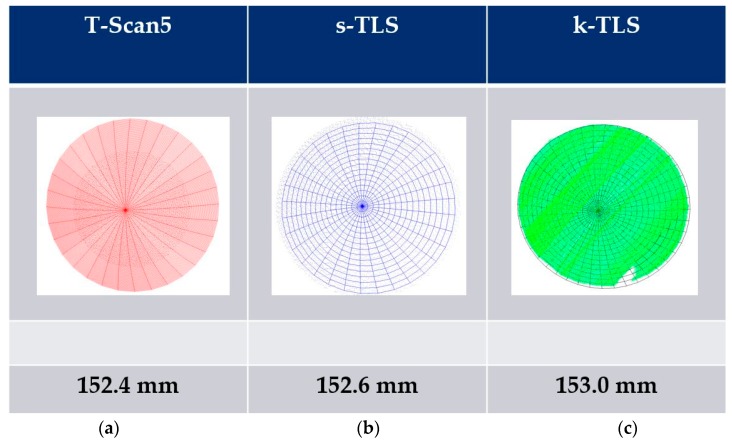
Focal length of paraboloid in mm derived from (**a**) T-Scan 5; (**b**) static and (**c**) k-TLS measurements calculated with Spatial Analyzer.

Three values for the focal length are determined (Figure 16). Referring to the reference value (derived from T-Scan 5 measurement) of 152.4 mm the resulting difference amounts to a deviation of −0.2 mm for s-TLS and −0.6 mm for k-TLS as a parameter for the precision.

For the measurements of a pillar (cylinder) the diameter was estimated with Leica Cyclone. According to the paraboloid three different diameters were calculated.

**Figure 17:**
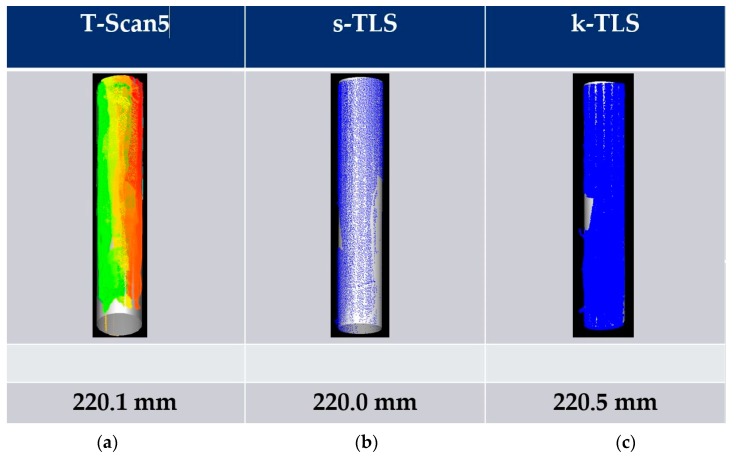
Diameter of a cylinder in [mm] derived from (**a**) T-Scan 5; (**b**) static TLS and (**c**) k-TLS measurements calculated with Leica Cyclone.

Referring to the reference value (derived from T-Scan 5 measurements) of 220.1 mm, the resulting difference amounts to a deviation of 0.1 mm for s-TLS and −0.4 mm for k-TLS (Figure 17) as a parameter for the precision.

### 5.2. Cloud to Cloud Comparison Using the M3C2-Algorithm

In addition to the analysis of geometrical parameters, a direct point cloud to point cloud analysis based on the M3C2- algorithm described in [31] was performed for the comparison of s-TLS to T-Scan 5, k-TLS to T-Scan 5 and s-TLS to k-TLS. This process was carried out for all reference geometries. The results for the comparison of s-TLS and k-TLS to T-Scan 5 for the cylinder, the curved surface and the cuboids are shown in this section. In this case the comparison is based on the same data used for the comparison of the geometrical parameters shown in Section 5.1.

**Figure 18:**
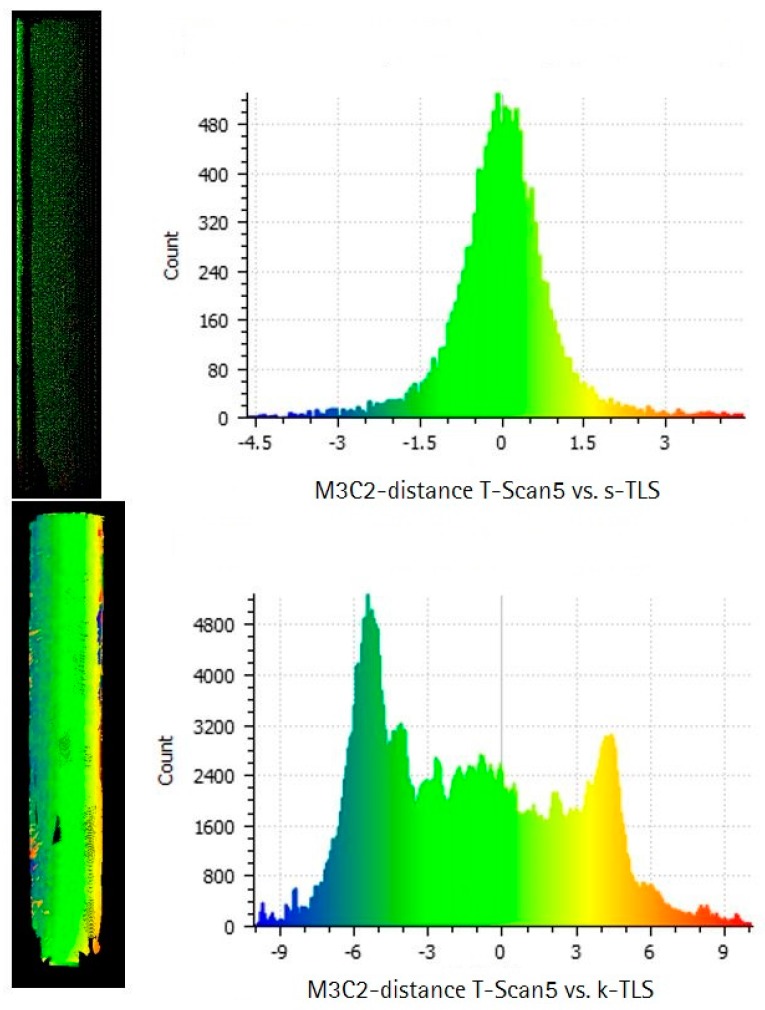
M3C2-distance in mm calculated with Cloud Compare for the cylinder, (**right**) difference point cloud and (**left**) histogram for T-Scan 5 compared to (**top**) s-TLS (**top**) and (**bottom**) k-TLS.

The cloud to cloud comparison of the cylinder for T-Scan 5 and s-TLS (Figure 18, top) shows approximately a normal distribution with a bias of −0.3 mm with 95% of the differences in a range of ±1.5 mm.

Although the comparison of the cylinders diameter amounts to a deviation of −0.4 mm for k-TLS the M3C2- algorithm provides an accuracy of −6 mm and +6 mm at the opposite edges of the cylinder (Figure 18, bottom). This indicates an error in the referencing of the measurement pillar caused by an incorrect synchronization or calibration of the MSS. Errors in calibration/synchronization of the MSS leads to an incorrect allocation of scan profiles and position data supplied by laser tracker and T-Probe, resulting in a shift of the geometries position along the trajectory of the MSS.

**Figure 19:**
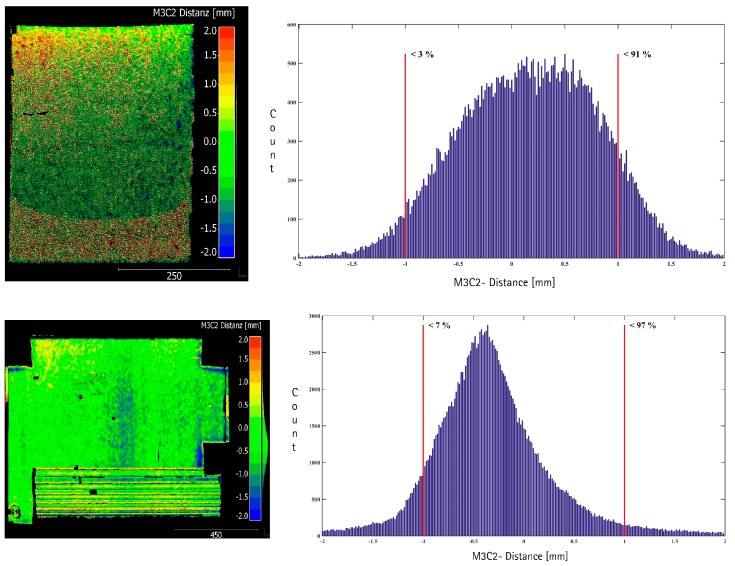
M3C2-distance [mm] calculated with Cloud Compare T-Scan 5 compared to (**top**) k-TLS for curved surface and (**bottom**) cuboids, Modified from [5].

Figure 19 (top) shows the point cloud to point cloud difference of the curved surface between the k-TLS-based MSS and the reference measurements with the Leica T-Scan 5. The histogram of the differences (Figure 19, top right) shows a range of −2 mm to +2 mm with a bias of approximately 0.3 mm. Around 90% of the differences are less than 1 mm. The maximum differences with up to +2 mm are shown in the upper and lower range of the reference geometry. The mean range is characterized by a homogeneous distribution of the differences. A clear systematic distribution of the differences with respect to the different angles of incidence for the range from about 30 gon to about 100 gon can’t be determined.

Figure 19 (bottom) shows the point cloud to point cloud difference for the cuboids between the k-TLS-based MSS and the reference measurements with the Leica T-Scan 5. The histogram shows a concentration in the range of −1.2 mm to +0.6 mm and a slightly left-leaning distribution. Approximately 94% of the differences are less than 1 mm (Figure 19, bottom right). At the horizontal edges of the cuboids the differences grow to +2.0 mm, due to the flat angle of incidence, as well as the laser spot size and the associated “mixed pixel” characteristics at the edges of the cuboids. The metallic cuboids show isolated data gaps, which results from adverse reflections. The bottom cuboid of wood, shows differences with a magnitude of 3 mm. Due to its edge length of 5 mm, this cuboid can’t be reliably detected.

## 6. Conclusions and Outlook

A complete framework for the quality analysis of geodetic sensors and MSS was discussed and presented in a general form and exemplarily. This framework, consisting of a laboratory, calibrated targets, a reference frame and geometries is the required infrastructure and key point for the backward modelling of quality measures and parameters. It should be adoptable for nearly all kinds of (geodetic) sensors. For two specific scenarios, s-TLS and k-TLS first results, determining accuracy and precision, are presented. To perform a backward modelling, additional evaluations based on the sampled data have to be implemented. That means no further data has to be collected. To examine and identify effects of the mentioned influencing factors the described evaluations have to be supplemented by additional approaches with varying distances and angles of incidence. One, if not the most crucial, role for a MSS is the sensor integration, where the temporal and spatial referencing of the sensors involved is to be determined with the utmost care in order to keep possible effects from remaining calibration deviations as low as possible. First results show that the presented framework is suitable to detect uncertainties and deviations in the calibration and synchronization of the MSS.

## Figures and Tables

**Table 1:** Environment for the calibration of geodetic sensors and MSS.

Component	Functions	Requirements
Laboratory	Environment for reference frame and reference geometries.	Stable environmental conditions (temperature, atmospheric pressure, humidity), geometric stability of the reference points.
Reference frame	Reproducibility of the measurements, common reference frame for the participating sensors, referencing of the sensors and geometries.	Calibrated targets representing the reference points, targets must be measurable by all participating sensors (point based, area based sensors), reference points must be stable, coordinates of the reference points with higher accuracy than tested sensors, reference points have to be well distributed.
Reference geometries	Investigation of different influence factors (distance, incident angle, material, colour) for point based and area based sensors.	Different materials (wood, metal, plastic), different colours (white, …, black), different dimensions (point, plane, sphere), different shapes and curvatures.
Reference sensors	Providing data with superordinate accuracy.	Higher level of accuracy than the tested sensors (sub mm).
Investigated sensors/MSS	Sampling reference geometries (volume, area or point based).	No special requirements.

**Table 2:** Parameters of the reference geometries and the sensitive influence factors that can be detected.

Reference Geometry	Shape	Material	Colour	Sensitive Influence Factors
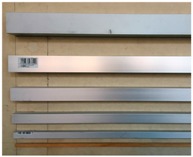	cuboids	5× metal, 1× wood	silver, light brown	resolution
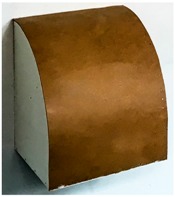	curved surface	wood	dark brown	incidence angle (vertical)
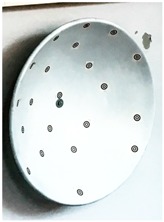	paraboloid	metal	white	incidence angle (horizontal and vertical)
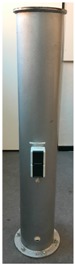	cylinder	metal	silver	incidence angle (horizontal), synchronization, referencing
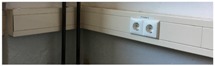	cuboid (cable channel)	plastic	grey	distance, incidence angle (horizontal and vertical)

**Table 3:** Accuracies of the participating sensors (according to manufacturer specifications).

Sensor	Accuracy
Leica AT960 LR	±15 µm + 6 µm/m (angle accuracy, maximum permissible error (MPE))
	±0.5 µm/m (distance accuracy Absolute Interferometer, MPE)
Leica T-Probe	U_x,y,z_ = ±30 µm + 10 µm/m uncertainty up to 25m, (MPE)
Leica T-Scan 5	U_x,y,z_ = ±60 µm under 8.5 m (MPE)
	UP = ± 80 μm + 3 μm/m (2σ, uncertainty for plane surfaces)
Z+F Imager 5006	0.007° rms (angle horizontal and vertical)
	1.2 mm rms (distance noise, 10 m, reflectivity black)
	0.7 mm rms (distance noise, 10 m, reflectivity dark grey)
	0.4 mm rms (distance noise, 10 m, reflectivity white)

**Table 4:** Number of points for reference geometries scanned with T-Scan 5.

Reference Geometrie	Point Cloud (T-Scan 5)	Number of Points
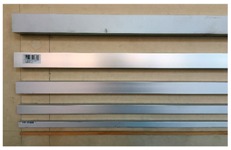	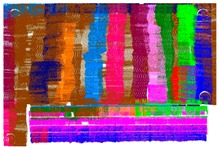	12,500,000
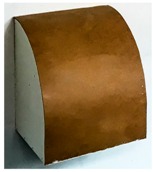	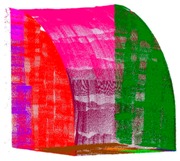	6,500,000
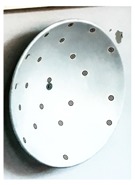	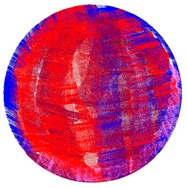	7,500,000
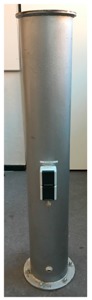	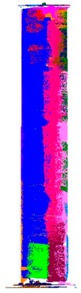	17,000,000
